# The Impact of Small Bowel Endoscopy in Patients with Hereditary Hemorrhagic Telangiectasia

**DOI:** 10.4274/tjh.2018.0253

**Published:** 2018-11-13

**Authors:** Stefania Chetcuti Zammit, David S. Sanders, Mark E. McAlindon, Reena Sidhu

**Affiliations:** 1 Sheffield Teaching Hospitals, Royal Hallamshire Hospital, Academic Department of Gastroenterology, Sheffield, England

**Keywords:** Hereditary hemorrhagic telangiectasia, Small bowel capsule endoscopy, Argon plasma coagulation

## To the Editor,

We have read with interest the article entitled “Thalidomide for the Management of Bleeding Episodes in Patients with Hereditary Hemorrhagic Telangiectasia: Effects on Epistaxis Severity Score and Quality of Life” [1].

This article highlights the use of thalidomide in the management of patients with hereditary hemorrhagic telangiectasia (HHT) who present with epistaxis. The prevalence of HHT is thought to be between 1.5 and 2 cases per 10,000 people [2]. HHT can be associated with other bleeding complications such as bleeding from the gastrointestinal tract and in particular the small bowel (SB). The existence of small bowel angioectasias (SBAs) has been reported to vary between 56% and 91% in the literature [3,4,5,6]. The study by Ingrosso et al. [6] also reported that patients with SBAs were considerably older.

We carried out a study at our tertiary center for the management of patients with HHT where 10 patients (60% males) with genetically confirmed HHT were referred for the management of gastrointestinal-related complications. The impact of small bowel capsule endoscopy (SBCE) and double balloon enteroscopy (DBE) was evaluated. The mean age at first SB endoscopy was 62.6±14.4 years (mean ± standard deviation).

Patients had a total of 39 gastroscopies, 16 colonoscopies, and 6 push enteroscopies. Seven patients underwent SBCE: 6 (85.7%) had proximal, 1 (11.1%) had mid, and 3 (33.3%) had distal SBAs. Two patients had a colon capsule that showed angioectasias.

Several DBEs were carried out for 6 patients (median 4; SD ±6) with a mean of 130.5±133.3 days between DBEs. Fifty-seven SBAs were treated with argon plasma coagulation (APC) on average at each DBE. These procedures take an average of 75 minutes. Mean hemoglobin before and after the procedure was 9.8 and 10.2 g/dL, respectively (p=0.1). Six patients were transfusion-dependent initially but 4 improved following intervention.

Need for transfusion resolved in 1 patient when started on lanreotide (a long-acting somatostatin analog), regular endoscopy, and APC, and in 2 patients upon starting DBEs and APC. One patient passed away from pneumonia. Another patient was switched unsuccessfully from octreotide to lanreotide. She stopped being transfusion-dependent with regular gastroscopies and APC. Another patient was unwilling to undergo further endoscopies due to multiple comorbidities. He improved on lanreotide. In 2 patients, anemia remains persistently problematic. One of them is also on dalteparin for superior mesenteric venous thrombosis. The other patient has recurrent epistaxis, which makes it harder for him to have further endoscopies.

SBCE is a useful screening tool in patients with HHT to assess SBAs. Although classed as invasive endoscopy, DBEs and APC can have a significant impact on mortality and quality of life in patients with HHT. Pharmacotherapy such as somatostatin analogs can additionally help to improve transfusion requirements. They have a good safety profile [7], unlike thalidomide, which can result in teratogenicity [8], peripheral neuropathy (50%) [9], and thromboembolism [10].
